# Fracture severity of distal radius fractures treated with locking plating correlates with limitations in ulnar abduction and inferior health-related quality of life

**DOI:** 10.3205/iprs000099

**Published:** 2016-07-28

**Authors:** Serafim Tsitsilonis, David Machó, Sebastian Manegold, Björn Dirk Krapohl, Florian Wichlas

**Affiliations:** 1 Charité – University Medicine Berlin, Center for Musculoskeletal Surgery, Berlin, Germany; 2 Department for Plastic Surgery and Hand Surgery, St. Marien Hospital, Berlin, Germany

**Keywords:** distal radius fracture, locking plate, functional outcome, loss of reduction, quality of life

## Abstract

**Introduction/background:** The operative treatment of distal radius fractures has significantly increased after the introduction of locking plates. The aim of the present study was the evaluation of health-related quality of life, functional and radiological outcome of patients with distal radius fractures treated with the locking compression plate (LCP).

**Materials and methods:** In the present study 128 patients (130 fractures) that were operatively treated with the LCP (2.4 mm/3.5 mm, Synthes^®^) were retrospectively evaluated. Mean follow-up was 22.7 months (SD 10.6). The fractures were radiographically evaluated (radial inclination, palmar tilt, ulnar variance) pre-, postoperatively and at the last follow-up visit. Range of motion (ROM) was documented. Grip strength was assessed with the use of a JAMAR dynamometer. The score for disabilities of the arm, shoulder and hand (DASH) and the Gartland-Werley score (GWS) were evaluated. Health-associated quality of life was assessed with use of SF-36 Health Survey.

**Results:** Postoperative reduction was excellent; at the last follow-up visit only minimal reduction loss was observed. Except for pronation, a statistically significant decrease of ROM was present; in most cases that was not disturbing for the patients. The injured side achieved 83.9% of grip strength of the intact side. Mean DASH was 18.9 and mean GWS was 3.5. Health-associated quality of life was generally not compromised. However, limitations in ulnar abduction correlated with inferior quality of life. Fracture severity correlated with inferior quality of life, despite the absence of correlation with the functional and radiological outcome. Complication rate was low.

**Conclusions:** Fracture severity seems to affect ulnar abduction and therefore patient quality of life, despite almost anatomical reduction; the objective and subjective scores were in most cases excellent. Modern everyday activities, such as keyboard typing, could be associated with the present results.

## Zusammenfassung

**Einleitung:** Die operative Therapie der distalen Radiusfrakturen hat nach der Entwicklung der winkelstabilen Platten signifikant zugenommen. Ziel der vorliegenden Studie war die Evaluation von Lebensqualität sowie funktionellem und radiologischem Outcome von Patienten nach einer winkelstabilen Plattenosteosynthese bei einer distalen Radiusfraktur.

**Materialien und Methoden:** Insgesamt wurden 128 Patienten (130 Frakturen) nach operativer Versorgung mittels Locking Compression Plate (LCP) (2,4 mm/3,5 mm, Synthes^®^) retrospektiv untersucht. Die mittlere Nachbeobachtungszeit betrug 22,7 Monate (SD: 10,6). Die Frakturen wurden prä-, postoperativ und bei der letzten Vorstellung radiologisch evaluiert (radiale Inklination, palmarer Tilt, Ulnavarianz). Der Bewegungsumfang wurde erhoben. Griffkraft wurde mittels eines JAMAR-Dynamometers dokumentiert. Der Disabilities of the Arm, Shoulder and Hand (DASH)-Score und der Gartland-Werley-Score wurden erhoben. Die gesundheitsassoziierte Lebensqualität wurde mittels des SF-36-Health-Survey evaluiert.

**Ergebnisse:** Die postoperative Reposition war exzellent; ein minimaler Repositionsverlust lag bei der letzten Follow-up-Vorstellung vor. Es wurde in jeder Bewegungsrichtung (außer Pronation) eine statistisch signifikante Minderung der Beweglichkeit beobachtet; in den meisten Fällen war dies nicht klinisch relevant. Die verletzte Seite erreichte 83,9% der Griffkraft der Gegenseite. Der Mittel-DASH-Score betrug 18,9 und der Mittel-GWS war 3,5. Die Lebensqualität war insgesamt nicht beeinträchtigt. Allerdings korrelierte die Einschränkung der Ulnarabduktion mit schlechteren Werten der Lebensqualität. Der Schweregrad der Fraktur korrelierte mit schlechterer Lebensqualität, trotz der fehlenden Korrelation mit dem funktionellen und radiologischen Outcome. Die Komplikationsrate war niedrig.

**Schlussfolgerungen:** Der Schweregrad der Fraktur scheint die Ulnarabduktion zu beeinflussen und dadurch die Lebenqualität zu beeinträchtigen, trotz der anatomischen Reposition. Alltagsaktivitäten, wie die Benutzung der Computertastatur könnte die Erklärung dafür sein.

## Introduction

The distal radius fracture is the most common extremity fracture overall with an annual incidence of 20/10.000 worldwide, meanng 10–25% of all fractures [1]. Several treatment modalities are available nowadays. Traditionally, distal radius fractures were conservatively treated by closed reduction and plastering. However, an increasing number of distal radius fractures are treated nowadays through open reduction and internal fixation with the use of locking plates, mostly through a palmar approach [2]. A certain degree of controversy still remains in terms of complications, technical challenges and the relation between radiological and functional outcome [3], [4]. While some support the notion that anatomical reduction should be the cornerstone of treatment [5], others have shown that anatomical reduction does not need not be necessarily achieved at all costs [6]. Conflicting results also exist in the literature, as far as complication rates and especially tendon ruptures are concerned [3], [7], [8]. 

Under this scope, the aim of the present study was to evaluate the functional and radiological outcome of patients with distal radius fractures treated operatively with a locking compression plate (LCP) in correlation to fracture severity and loss of reduction and examine complication rates and health-associated quality of life.

## Materials and methods

All distal radius fractures that were not suitable for conservative treatment and were operatively treated with locking plating (2.4 mm and 3.5 mm LCP, Synthes^®^, Umkirch, Germany) over a period of two years (2008–2009) in our institute, were retrospectively evaluated. The study was approved by the local ethics committee (EA2/075/11). Study population consisted of 130 distal radius fractures (128 patients) with a mean follow-up period of 22.7 months (SD 10.6 months, median 19.7 months). Mean population age was 58.1 (SD 16.4/range 17–92). The majority of the patients were women (n=85/65.4%). The left side was fractured more often (n=74/56.9%). Recorded data were: radial inclination, palmar tilt and ulnar variance pre-, postoperatively and at last follow-up visit, AO/OTA fracture classification, implant type, complications and implant removal. Active range of motion (ROM) and grip strength with a JAMAR dynamometer (Model 5030J1, Sammons Preston, USA) were examined. The contralateral side served as control. Patients with an injury of the contralateral hand in the past that had resulted in cast immobilisation for more than 3 weeks were excluded from comparison of ROM and grip-strength (n=29). Fractures were radiographically evaluated with posto-anterior (p.a.) and lateral radiographs according to the criteria of Kreder et al. [9]. Objective hand function was assessed with the Gartland and Werley score (GWS) [10] and subjective function with the score for disabilities of the arm, shoulder and hand (DASH) [11]. Health-associated quality of life was assessed with SF-36-health-survey questionnaire [12]. 

### Fractures

The major mechanism of injury was fall from standing height (n=93/71.5%). Other mechanisms were fall from greater height (n=17/13%), road accident (n=14/10.8%) and sport accident (n=6/4.7%). Fractures were classified according to AO by two independent trauma surgeons. More than half (n=70/53.8%) were type C fractures. Fractures were divided in two groups, depending on surgical approach (palmar/dorsal). Palmar approach was performed in 93 cases (71.5%); dorsal in 34 fractures (26.2%); in 3 fractures a combined dorsopalmar approach was used (2.3%). Except for one, all fractures were closed. In 82 cases (63.1%) fractures were accompanied by grade II soft-tissue injuries according to Tscherne and Oestern, while in 46 cases (35.4%) by grade I a 2.4 mm LCP was used in 94 cases (72.3%), a 3.5 mm LCP in 33 cases (25.4%). In three cases both plates were used. The surgical approaches are described in detail elsewhere [13]. 

### Statistical analysis

Continuous variables were expressed as means ± standard deviation (SD); categorical variables as percentages (%). Kolmogorov-Smirnov test was used for normality assessment of distributions. For parametric continuous variables Student t-test was used for comparison of two groups, while for non-parametric Wilcoxon signed-rank test for paired or Mann-Whitney U-test for independent data was implemented. Differences for categorical variables were assessed with χ^2^-test or Fisher’s exact test. Correlations were examined with either Pearson product moment correlation coefficient or Spearman’s rank correlation coefficient. Differences were considered as statistically significant if the null hypothesis could be rejected with >95% confidence (p<0.05).

## Results

**Figure 1:**
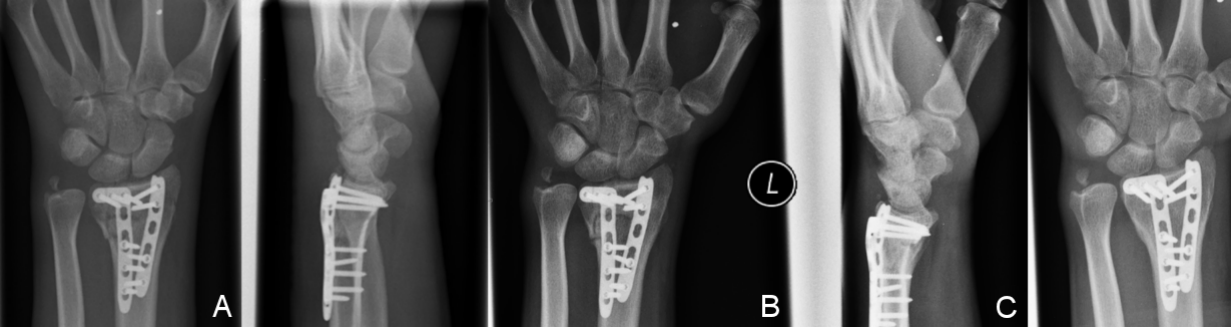
Radiographic follow-up series of a 46-year-old female patient with an AO 23C3 fracture, treated through a dorsal approach with 2.4 mm LCP (Synthes^®^). A) Postoperative x-ray with excellent reduction. B) Follow-up radiographs after 12 weeks. C) Follow-up radiograph one year after trauma with excellent radiological fracture consolidation and no significant loss of reduction.

**Figure 2:**
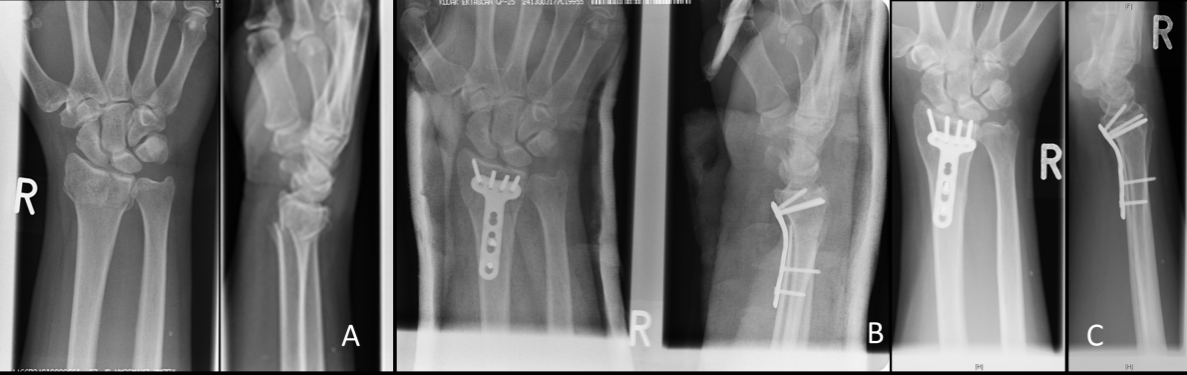
Radiographic follow-up series of a 52-year-old female patient with an AO 23C2 fracture, treated through a volar approach with 2.4 mm LCP (Synthes^®^). A) Posttraumatic x-ray. B) Follow-up radiographs after 12 weeks. C) Follow-up radiograph 16 months after trauma with no major loss of reduction.

**Figure 3:**
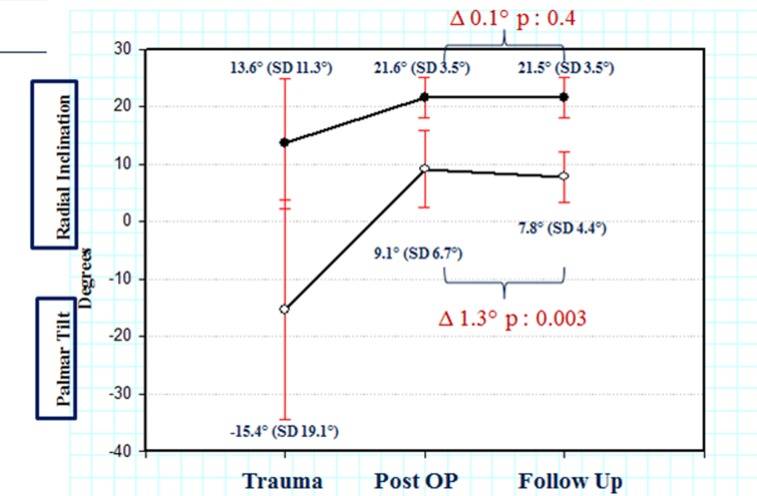
Radiological values of radial inclination and palmar tilt pre-, postoperatively and at the last follow-up visit. A very good reduction was achieved postoperatively with an excellent retention at the last follow-up visit.

The postoperative fracture reduction was excellent (Figure 1 (Fig. 1) and Figure 2 (Fig. 2)). A minimal loss of reduction was observed at the last follow-up visit (Table 1 (Tab. 1), Figure 3 (Fig. 3)); the difference in palmar tilt was statistically significant. ROM impairment was seen in all planes. Flexion was mostly affected. Differences were statistically significant for all planes, except for pronation (Table 2 (Tab. 2)). No correlation between ROM and surgical approach was observed. A statistically significant decrease in grip strength was observed. The fractured side achieved 83.9% of grip strength of the intact side. Mean DASH was 18.9 (SD 21.1); this is a normal value compared to the DASH of the age group between 50–65 years of the normal German population. DASH increased with age. Mean GWS was 3.5 (SD 4); most patients showed excellent objective function and 85.3% of the patients showed excellent to good results. Evaluation of mean health-associated quality of life with SF-36 Health Survey compared to the age group between 50–59 years of the normal population revealed that general health perception was not greatly affected (Table 3 (Tab. 3)).

In 26 cases (20.3%) intermittent mild pain, mostly weather-associated, was evident. Seven patients presented with intermittent paraesthesias of the wrist and in seven cases with persistent swelling. Patients with type C fractures according to AO classification suffered significantly more complications (15%) compared to patients with other types of fractures (3.5%, p<0.001). Nevertheless, the final functional outcome did not differ between those two groups. Neither deep infections, nor non-unions were observed. There was no difference in tendon-associated complications between surgical approaches. In six cases tendon-associated complications were observed that led to implant removal. In three cases tenosynovitis of finger flexors was observed, while in other three cases tenosynovitis of the extensor pollicis longus muscle tendon. Symptoms subsided after implant removal. No tendon ruptures were observed. 

Fracture severity after AO/OTA classification negatively correlated with ulnar abduction (p=0.022, Spearman’s rho: –0.205). Limitation in ulnar abduction was the only parameter of ROM that correlated with inferior quality of life in many subcategories of SF-36 (Table 4 (Tab. 4)). There was no correlation between fracture severity according to AO classification and functional outcome, as assessed through DASH and GWS. A weak negative correlation between ROM (flexion/extension arch) and age was observed (p=0.001, Pearson’s r: –0.284). The correlation was stronger for male gender (p=0.028, Pearson’s r: –0.328). Fracture severity weakly correlated with operative time (p=0.05, Spearman’s rho: 0.246). There was no correlation between fracture severity and loss of reduction.

## Discussion

In the present study we show that patients with distal radius fractures treated with LCP have overall good functional and radiological outcome, with low complication rates and good health-associated quality of life. However, fracture severity still correlated with limitations in ulnar abduction and inferior health-related quality of life. 

It was interesting to observe this correlation between fracture severity and quality of life. AO classification has been traditionally thought to bear prognostic value on the final outcome. The evaluation of the outcome of distal radius fractures in general, without stratification for different types of treatment, confirms this fact [14]. However, there seems to be an inconsistency in the case of distal radius fractures treated with locking plating. The radiological and overall functional outcome, as evaluated with the DASH and GWS, show very good results in the present study. Nevertheless, limitations in ulnar abduction seem to deteriorate the quality of life of those patients. Simple activities of daily living, such as opening a bottle or typing on a keyboard, seem to be affected in cases of severe fractures in the long term. The importance of ulnar abduction seems to be underestimated in many studies. Additionally, the reported scores also do not seem to reflect the limitations in every-day life and the resulting limitation in quality of life of those patients. Souer et al. could not demonstrate significant differences in ROM and functional scores between patients with intra-articular and patients with extra-articular radius fractures at any of the follow-up time points [15]. Additionally, Konstantinidis et al. did not observe differences in functional scores between type Type C1 and C3 fractures [16]. That seems to be also the case for palmar multidirectional fixed-angle plate fixation, with intra-articular fractures not having a worse outcome than extra-articular ones one year after trauma [13]. Grip strength also did not correlate with fracture severity in other studies [17]. 

The reasons for this finding are not absolutely clear. It would be unwise to simply suggest that AO classification has neither prognostic relevance nor importance, as it can be shown in the present study. A combination of treatment- and injury-specific factors seems more probable. One inherent problem of the classification might be the fact that simple extra-articular fractures with a non-displaced intra-articular split are classified as type C fractures, whereas in terms of surgical technique and outcome resemble AO type A fractures. Furthermore, even when intra-articular fracture lines result in structural arthritic changes, they do not necessarily mean inferior outcome in the case of distal radius fractures. In the study of Catalano et al. 76% of the patients at an average of 7.1 years postoperatively showed arthritic changes; nevertheless, functional outcome was not impaired [18]. Finally, plate design itself could also attribute to the observed results. Pre-bent plates offer better handling modalities and in the hands of experienced surgeons can result in almost anatomic reduction without major additional irritation of the surrounding tissues [15].The radiological examination in our study with the highly satisfying postoperative reduction confirms this. As the plate is anatomically pre-contoured, it can be used for fracture reduction or to reduce the fracture to the plate; a good plate- and screw-positioning is an indicator for correct reduction. On the other hand, the majority of the patients with inferior quality of life in the present study showed a fractured ulnar process that was not addressed osteosynthetically. The importance of this finding remains to be evaluated. 

The excellent biomechanical behavior of locking plates was also confirmed, as only minimal reduction loss was seen at the last follow-up visit. Despite the statistically significant difference in palmar tilt, the absolute value of 1.3° was small. This minimal loss of reduction could be attributed to the locking principle of the screws that buttress the joint surface. Especially the 2.4 mm LCP permits placement of up to 5 locking screws for the joint surface, thus addressing single fragments. These results are comparable to previous studies, where radiological parameters were successfully reduced [7], [19]. Konstantinidis et al. observed a loss of reduction of 1° in the palmar tilt, while radial inclination and ulnar variance remained almost unaffected [16]. In the present patient collective the distal screws were monocortically fixed; the minimal loss of reduction is indicative of the good fixation achieved by locking plating without the need of bicortical screws that can increase tendon irritation risk [20], [21]. Whether a loss of reduction could be absolutely prevented is rather questionable. Theoretically, ideal screw placement is located subchondrally, as near to the joint as possible. However, placing the plate distally to the water-shed line, leading to tendon-associated problems, and the increased risk of intra-articular screw placement set practical limits. Recent studies have questioned the importance of anatomical reduction of distal radius fractures [6]. We believe that anatomical reduction can support good functional outcome; however, it should not be achieved at all costs, as it does not seem to be the only prerequisite for adequate function. Meticulous surgical soft-tissue handling and postoperative rehabilitation could also affect outcome. 

Despite excellent fracture reduction, a statistically significant ROM decrease in almost every plane was observed with flexion being mostly affected. Previous studies have shown greater ROM decrease in comparison to our results [22]. In the study of Knight et al. a ROM decrease of 25% was seen, except for supination and pronation, with flexion being also mostly affected [7]. That was also the case in the study of Matschke et al. [17]. This decrease in flexion was attributed to scar tissue formation over the palmar plate that could hinder tendon motion. Additionally, active flexion postoperatively is probably what hurts the most, due to the approach, and is therefore least performed by the patients.

Supination and pronation were also the least affected in previous studies, as was the case in our patient collective. An explanation for this could be that supination and pronation occur in the distal radioulnar joint, which in many distal radius fractures, such as type A and most type C factures, remains almost intact [23].

The subjective outcome, as evaluated with the DASH, was satisfying compared to the normal general german population (DASH score: 13, SD 15) [12]. The comparison of DASH score of the study population with a mean age of 58.1 years with that of the respective age group of the normal general population (19.0, SD 18) shows good subjective patient satisfaction. Our study shows higher DASH values compared to previous studies. However, this could be attributed to region-specific population characteristics, as the normal population values indicate. 

The objective functional results as assessed by GWS were also good with a mean score of 3.5 points. This result is in accordance with previous studies [24]. It was interesting to see that no patients were observed with inadequate objective functional outcome, whereas almost 85% of the patients had excellent to good results. 

In terms of health-related quality of life, the results were overall satisfying. The scores for physical functionality and psychological wellness were 72.5 and 71.2 respectively, while in other studies they were not over 57 points [3]. This could be attributed to the low complication rates, as well as the satisfying objective and subjective scores. Nevertheless, even despite the absence of statistically significant differences between patients with type C3 and the remaining fractures, the observed correlation between fracture severity and inferior quality of life indicates that other factors (for instance fracture of the ulnar styloid process or even psychological parameters) could affect the quality of life of those patients. 

The overall complication rates are in also accordance with previous studies [15]. However, the high rate of tendon-associated complications previously reported was not confirmed [17], [22]. It was interesting to see that no tendon ruptures were observed. Patients that presented with tendon-associated problems were successfully treated with implant removal. The use of monocortical distal screws can minimize the risk of oversized screws that penetrate the opposite cortex and lead to tenosynovitis and eventually rupture of the extensor tendons. Monocortical screws result in sufficient biomechanical osteosynthetic behavior, mainly through the principle of subchondral buttressing [21]. Interestingly the dimensions of the plates did not play an important role, as no significant differences were observed between the 2.4 mm and 3.5 mm plates in terms of complications. This point needs further clarification in our opinion. For dorsal surgical approaches a close follow-up could be meaningful, in order to early identify patients that in risk for tendon rupture. 

In conclusion, locking plating of distal radius fractures can overall result in very good functional and radiological outcome with satisfying health-associated quality of life. Nevertheless, fracture severity still affects the health-related quality of life of those patients, mainly through limitations in ulnar abduction.

## Notes

**Level of evidence:** level IV, retrospective case study.

### Competing interests

The authors declare that they have no competing interests.

## Figures and Tables

**Table 1:**
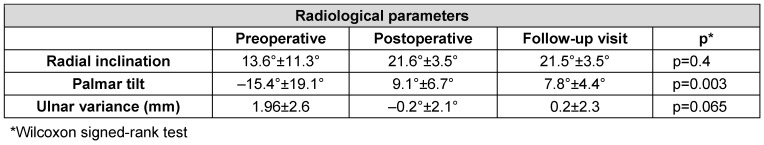
Radiological parameters of pre- and postoperative x-rays and of x-rays at last follow-up visit. The reduction of the fractures was excellent; only a minor loss of reduction was observed at the last follow-up visit.

**Table 2:**
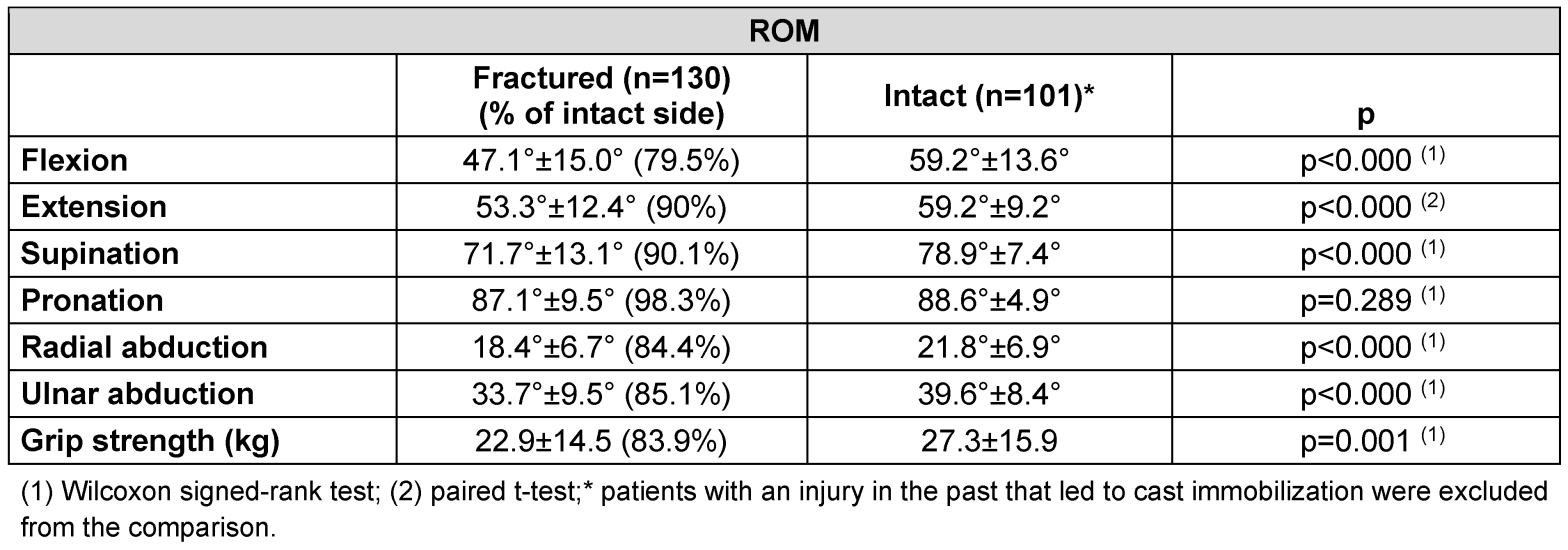
Range of Motion (ROM) between the fractured and the intact side. There was a statistically significant reduction of ROM in all directions except for pronation. The grip strength of the fractured side was statistically significantly lower.

**Table 3:**
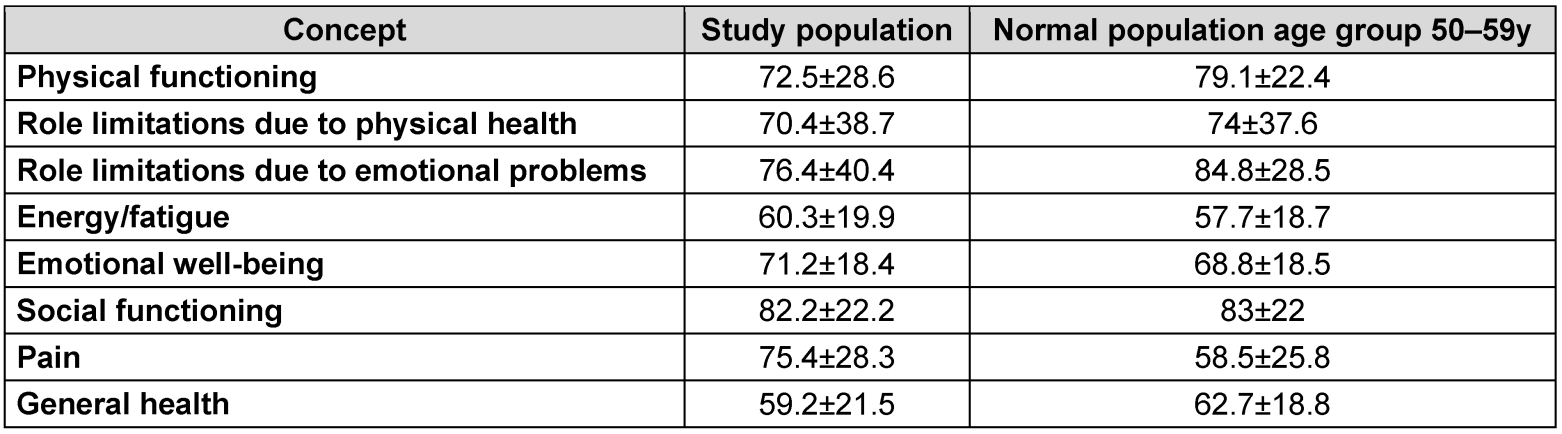
Scores of the different aspects of SF-36 of the study population and of the normal population age group 50–59 years

**Table 4:**
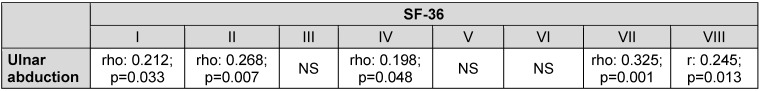
Correlations between ulnar abduction and subcategories of the health-related quality of life of the patients. (Physical functioning (I); Role limitations due to physical health (II); Role limitations due to emotional problems (III); Energy/fatigue (IV); Emotional well-being (V); Social functioning (VI); Pain (VII); General health (VIII))
